# Isolated Enteric Cyst in the Neck

**DOI:** 10.1155/2014/597813

**Published:** 2014-04-24

**Authors:** Amit Mahore, Raghvendra Ramdasi, Palak Popat, Shilpa Sankhe, Vishakha Tikeykar

**Affiliations:** ^1^Department of Neurosurgery, King Edward Memorial Hospital and Seth Gordhandas Sunderdas Medical College, Parel, Mumbai 400012, India; ^2^Department of Radiology, King Edward Memorial Hospital and Seth Gordhandas Sunderdas Medical College, Parel, Mumbai 400012, India; ^3^Department of Pathology, Bombay Hospital Institute of Medical Sciences & Research, New Marine Lines, Mumbai 400020, India

## Abstract

We report an extremely rare case of isolated enteric cyst in the neck region which was diagnosed on the histopathological examination. It was suspected to be duplication cyst on radiology. We have also evaluated the differential diagnosis and management issues.

## 1. Introduction

Endodermal cyst which is not connected to the nervous system or associated with vertebral abnormalities is called isolated enteric cyst [1]. Only one case of subcutaneous location of isolated enteric cyst has been reported in dorsal scapular region [2]. We report another case of similar subcutaneous cyst in the cervical region.

## 2. Case Report

A 32-year-old male, known case of neurofibromatosis type 2, presented with insidiously increasing painless swelling over left side of the neck over one year. On examination, a soft, spherical, nonpulsatile, nontransilluminant, and nontender swelling free from overlying skin and underlying structures was found in left anterior triangle of the neck. Computed tomography (CT) scan revealed well-defined isodense, nonenhancing mass in the left lateral aspect of the neck, medially abutting the left lateral wall of trachea and the oesophagus, laterally abutting the carotid sheath, and posteriorly abutting the vertebral body. However, no vertebral bony defect was noted in the spine (Figures 2(a) and 2(b)). For further characterisation of the lesion, magnetic resonance scan (MRI) of neck was done which showed the lesion to be hyperintense on T1, T2, and fat saturation sequences, suggesting a lesion with predominant proteinaceous or hemorrhagic component (Figures 2(c)–2(f)). On T2 weighted axial image, the lesion had a small beak-like extension (arrow in Figure 2(f)) towards the esophagus pointing to the radiaological diagnosis of esophageal duplication cyst. During surgery, the cyst was found to be free from all structures including esophagus and it contained mucoid viscous secretion. Total excision was done. His postoperative course was uneventful. Histopathological examination revealed the cyst wall to be lined by pseudostratified ciliated columnar epithelium with underlying smooth muscle bundle fibres arranged in two layers and lymphocytes in perivascular distribution. (Figures 3(a) to 3(d)). These findings are consistent with enterogenous cyst. At follow-up of 1 year, the patient has no recurrence.

## 3. Discussion

The differential diagnosis of cystic swelling in anterolateral neck includes branchial cleft cysts, thyroglossal cysts, thymic and thyroid cysts, dermoids, lymphangiomas, cystic hygromas, teratomas, cystic necrotic lymph node, foregut duplication cyst, enteric cyst, foregut cyst, and bronchogenic cyst [3]. Out of these, only the last four contain muscle in their walls and are lined by ciliated epithelium [1, 3].

**Figure 1:**
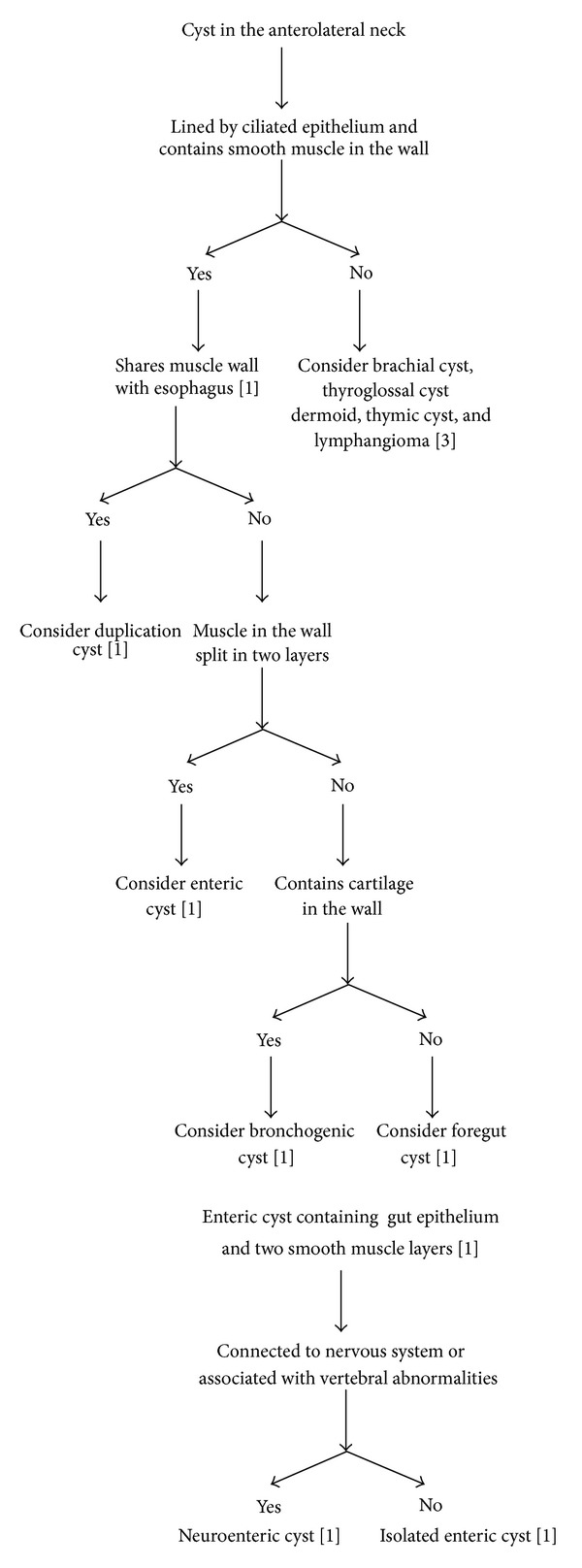


**Figure 2:**
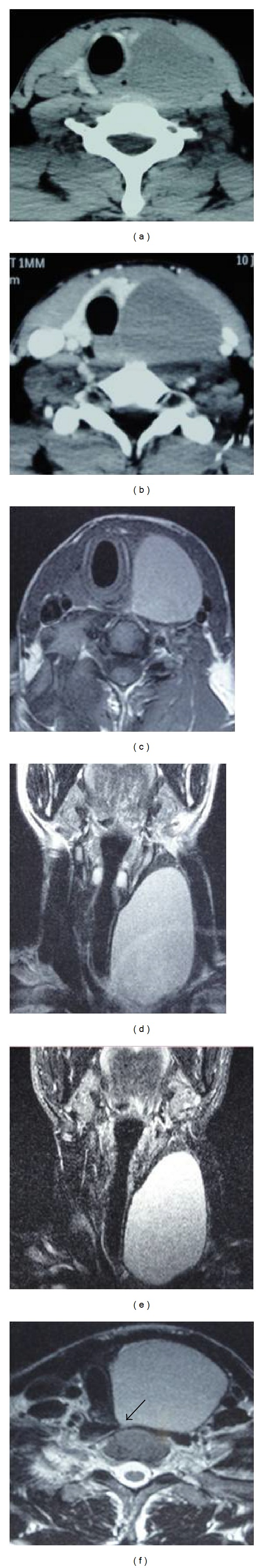
Plain (a) and contrast (b) axial images of computed tomography (CT) revealing isodense nonenhancing neck swelling. Axial images of T1 (c), T2 (f) weighted sequence, coronal images of T2 weighted (d), and fat saturation (e) sequences of magnetic resonance imaging (MRI) showing lesion to be hyperintense. On T2 weighted axial image, the lesion had a small beak-like extension ((black arrow in (f)).

**Figure 3:**
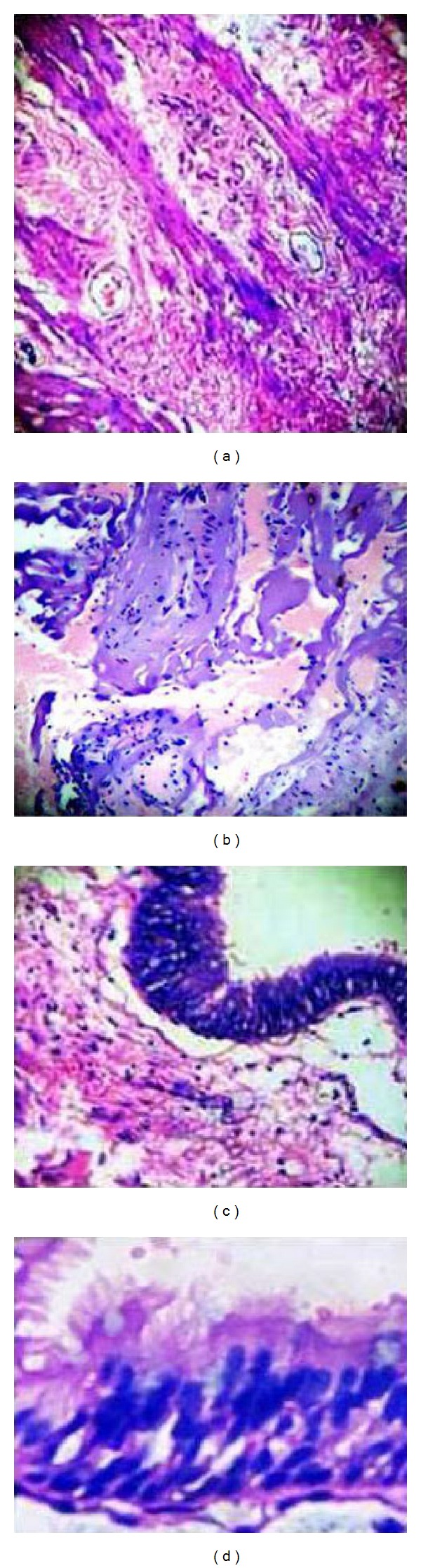
(a) and (b) are photomicrographs showing the cyst wall containing smooth muscle bundle fibres arranged in two layers: circular and longitudinal. (c) and (d) reveal the lining epithelium to be pseudostratified ciliated columnar (H&E; 400X).

Foregut duplication cysts and enteric cysts are derived from dorsal enteric portion of the foregut. Foregut cysts and bronchogenic cysts are derived from ventral respiratory portion of the foregut. The differentiation of duplication cyst from the rest of the cystic lesions is done according to the criteria laid down by Ladd and Grossa and later reinforced by Parker et al. These include (a) close proximity to the gastrointestinal tract, (b) lining which resemble some part of the gastrointestinal tract, and (c) an outer smooth muscle layer which either shares the muscle wall with the gut or is intermingled with the muscular coat of the bowel. Foregut and bronchogenic cysts do not have the two distinct layers of smooth muscle layers like enteric cysts. Bronchogenic cysts in addition have cartilage in their walls (Figure 1). In our case, cyst was completely isolated from esophagus and had two distinct smooth muscle layers in its wall, proving it to be isolated enteric cyst.

Isolated enteric cysts are neither connected to nervous system nor associated with vertebral abnormalities like neuroenteric cysts, both being histologically the same. These can migrate to different regions of the body. Only few cases outside the posterior mediastinum, which is the most common location, have been reported [1]. Four cases of abdominal, one in the testis, and one in the dorsal scapular region are described in literature [1, 2, 4]. Our case is the first case of isolated enteric cyst in the neck region and the second in the subcutaneous location.

Wilkens and Odom noted three histopathological types of enteric cysts. Type A cysts contain either columnar or cuboidal cells, with ciliated and nonciliated components. Type B cysts include all of the features of type A with addition of bone, cartilage, lymphatic tissue, fat, or glandular components. Type C cysts have type A features in association with ependymal or glial tissue [5]. Our Patient had isolated enteric cyst of type B.

Infection, spontaneous hemorrhage, and malignant transformation are reported complications of enteric cyst. Surgery remains primary modality of treatment as aspiration is associated with high recurrence. Even surgery has recurrence rate of 0–37% for which partial resection remains the primary risk factor. Long-term prognosis is excellent [6].

## 4. Conclusion

Isolated enteric cyst in the neck though extremely rare should be considered as a differential diagnosis of the neck swelling. Total surgical excision remains treatment of choice and the only way to prevent the recurrence.
